# Asaronic Acid Inhibited Glucose-Triggered M2-Phenotype Shift Through Disrupting the Formation of Coordinated Signaling of IL-4Rα-Tyk2-STAT6 and GLUT1-Akt-mTOR-AMPK

**DOI:** 10.3390/nu12072006

**Published:** 2020-07-06

**Authors:** Hyeongjoo Oh, Sin-Hye Park, Min-Kyung Kang, Yun-Ho Kim, Eun-Jung Lee, Dong Yeon Kim, Soo-Il Kim, Su Yeon Oh, Woojin Na, Soon Sung Lim, Young-Hee Kang

**Affiliations:** Department of Food Science and Nutrition and The Korean Institute of Nutrition, Hallym University, Chuncheon 24252, Korea; ohhyeongju@gmail.com (H.O.); SINHYEPARK@yuhs.ac (S.-H.P.); mitholy@hallym.ac.kr (M.-K.K.); royalskim@hallym.ac.kr (Y.-H.K.); reydmswjd@naver.com (E.-J.L.); ehddus3290@naver.com (D.Y.K.); ky4850@naver.com (S.-I.K.); suy0411@naver.com (S.Y.O.); nsm0729@hanmail.net (W.N.); limss@hallym.ac.kr (S.S.L.)

**Keywords:** asaronic acid, diabetes, glucose, interleukin-4, M2 macrophage, mTOR, STAT6

## Abstract

Macrophage polarization has been implicated in the pathogenesis of metabolic diseases such as obesity, diabetes, and atherosclerosis. Macrophages responsiveness to polarizing signals can result in their functional phenotype shifts. This study examined whether high glucose induced the functional transition of M2 macrophages, which was inhibited by asaronic acid, one of purple perilla constituents. J774A.1 murine macrophages were incubated with 40 ng/mL interleukin (IL)-4 or exposed to 33 mM glucose in the presence of 1-20 μΜ asaronic acid. In macrophages treated with IL-4 for 48 h, asaronic acid further accelerated cellular induction of the M2 markers of IL-10, arginase-1, CD163, and PPARγ via increased IL-4-IL-4Rα interaction and activated Tyk2-STAT6 pathway. Asaronic acid promoted angiogenic and proliferative capacity of M2-polarized macrophages, through increasing expression of VEGF, PDGF, and TGF-β. In glucose-loaded macrophages, there was cellular induction of IL-4, IL-4 Rα, arginase-1, and CD163, indicating that high glucose skewed naïve macrophages toward M2 phenotypes via an IL-4-IL-4Rα interaction. However, asaronic acid inhibited M2 polarization in diabetic macrophages in parallel with inactivation of Tyk2-STAT6 pathway and blockade of GLUT1-mediated metabolic pathway of Akt-mTOR-AMPKα. Consequently, asaronic acid deterred functional induction of COX-2, CTGF, α-SMA, SR-A, SR-B1, and ABCG1 in diabetic macrophages with M2 phenotype polarity. These results demonstrated that asaronic acid allayed glucose-activated M2-phenotype shift through disrupting coordinated signaling of IL-4Rα-Tyk2-STAT6 in parallel with GLUT1-Akt-mTOR-AMPK pathway. Thus, asaronic acid has therapeutic potential in combating diabetes-associated inflammation, fibrosis, and atherogenesis through inhibiting glucose-evoked M2 polarization.

## 1. Introduction

Macrophage polarization and functions are tightly controlled through the activation of various inter-connected pathways in several physiologic and pathologic states [1,2]. Activation of Janus kinase (JAK)-signal transducers and activators of transcription (STAT) downstream signaling is known to play an essential role in plasticity and heterogeneity of macrophages [1,3]. The STAT3/ STAT6 activation by interleukin (IL)-4, IL-13, and IL-10 induces M2 macrophage polarization, leading to tissue remodeling and repairing [4]. In addition, peroxisome proliferator-activated receptor γ (PPARγ) induces human monocytes toward an anti-inflammatory M2 phenotype. In fact, the balance between M1 and M2 macrophages is closely regulated in normal tissues and governs the fate of an organ in inflammation or injury [5]. The M1/M2 ratio increased during tissue inflammation process [6,7]. Since there is not a rigid barrier between macrophage phenotypes, it is difficult to classify macrophage subtypes in a proper and precise way [8]. Wound-healing macrophages produce elevated levels of growth factors such as platelet-derived growth factor (PDGF), insulin-like growth factor 1, vascular endothelial growth factor (VEGF) and transforming growth factor (TGF)-β1 [9,10], which aid in cellular proliferation, granulation, tissue formation, and angiogenesis. However, the diversity of macrophage phenotypes in vivo remains to be fully characterized.

Abundant literature establishes an important role for macrophage polarization in the pathogenesis of metabolic diseases [11,12]. There is an imbalance in the ratio of M1/M2 macrophages in obesity and type 2 diabetes, leading to chronic inflammation and the propagation of metabolic dysfunction [11]. Macrophages exposed to high glucose display M1 polarization, triggering podocytes apoptosis via activation of tumor necrosis factor-α-reactive oxygen species (ROS)-p38 mitogen-activated protein kinase pathway in diabetic nephropathy [13]. However, there is emerging evidence revealing that both M1 and M2 macrophages may coexist, leading to persistent inflammation and fibrosis in chronic inflammatory responses [14,15]. During the process of inflammation M1, macrophages initially elaborate pro-inflammatory cytokines and ROS, whereas the latter phase is controlled by M2 macrophages to resolve inflammation and promote tissue remodeling with release of growth factors [2,14]. Significant evidence shows the importance of M2 macrophage activation in both renal fibrosis and wound healing process of chronic kidney disease [16]. The mechanisms by which M2 macrophages induce renal repair are still under debate. M2 macrophages play crucial roles in determining short-term recovery following acute kidney injury [17,18].

**Figure 1:**
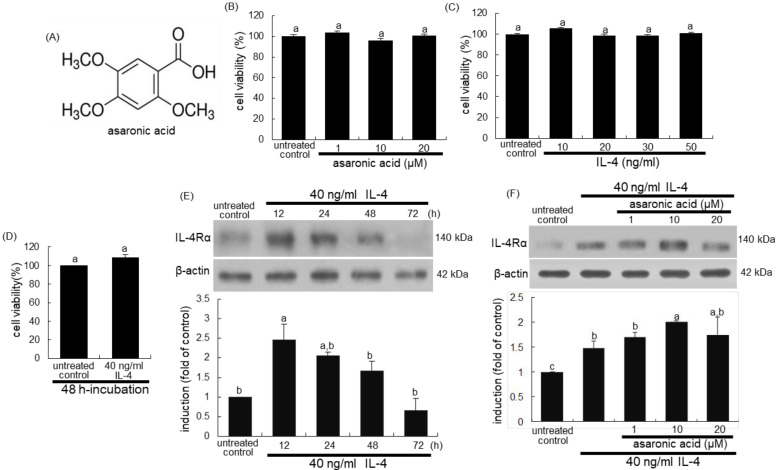
Chemical structure of asaronic acid (**A**), cytotoxicity of asaronic acid and IL-4 for up to 48 h (**B**–**D**), time-course response of IL-4 receptor IL-4Rα induction to IL-4 for up to 72 h (**E**), and further upregulation of IL-4Rα by asaronic acid (**F**). J774A.1 macrophages were exposed to 10-50 ng/mL IL-4 in the absence and presence of 1-20 μM asaronic acid for up to 72 h. Macrophage viability (mean ± SEM, *n* = 5) was measured by using 3-(4,5-Dimethylthiazol-2-yl)-2,5-diphenyltetrazolium bromide (MTT) assay and expressed as percent cell survival relative to untreated control (**B**–**D**). Cell lysates were subject to 8% SDS-PAGE and Western blot analysis with a primary antibody against IL-4Rα. β-Actin antibody was used as an internal control. The bar graphs (mean ± SEM, *n* = 3) in the panels represent quantitative results of the upper blot bands obtained from a densitometer. Mean values in respective bar graphs not sharing a same lower-case alphabet letter are significantly different at *p* < 0.05.

Several studies have shown the therapeutic potential of pharmacological regulators in the treatment of tissue injury through modulating macrophage polarization [19,20]. Recent studies highlighted several natural compounds that may possess the ability to regulate macrophage polarization [21,22,23]. For instance, the soya compound genistein induces macrophage polarization of M1 towards M2 macrophages and reduces systemic cytokines to ameliorate experimental colitis [24]. On the contrary, corosolic acid inhibits the M2 polarization of macrophages and tumor cell proliferation by blocking both STAT3 and nuclear factor (NF)-κB activation [24]. Our recent study showed that asaronic acid (2,4,5-trimethoxybenzoic acid, Figure 1A), newly identified in purple perilla extracts [25], hampered diabetic activation of macrophages toward the M1 phenotype through inhibiting NF-κB/JAK2-STAT signaling [26]. Nevertheless, the bioactive properties of asaronic acid have been seldom reported. The present study examined whether asaronic acid manipulated M2 macrophage phenotypes in diabetic macrophages. To induce M2 macrophages in the absence and presence of asaronic acid, J774A.1 murine macrophages were exposed to the M2 inducer IL-4 or 33 mM glucose for comparison. Furthermore, this study attempted to explore metabolic alterations required for M2 macrophage activation in IL-4-exposed or glucose-loaded macrophages. Asaronic acid may have therapeutic potential in counteracting diabetes-associated macrophage dysfunction through inhibiting glucose-evoked M2 polarization.

## 2. Materials and Methods

### 2.1. Materials

Dulbecco’s Modified Eagle Medium (DMEM) chemicals, RPMI 1640 medium, fatty acid-bovine serum albumin (BSA), and phorbol 12-myristate13-acetate (PMA) were provided by Sigma Aldrich Chemical (St. Louis, MO, USA), as were all other reagents, unless specifically stated elsewhere. 3-(4,5-Dimethylthiazol-2-yl)-2,5-diphenyltetrazolium bromide (MTT) was purchased from Duchefa Biochemie (Haarlem, Netherlands). Fetal bovine serum (FBS) and penicillin-streptomycin were obtained from Lonza (Basel, Switzerland). IL-4 protein and antibodies of PDGF and VEGF were purchased from R&D System (Minneapolis, MN, USA). Asaronic acid was purchased from Cayman Chemical (Ann Arbor, MI, USA). Antibodies of IL-4 receptor Rα (IL-4Rα), carbohydrate kinase-like (CARKL), cyclooxygenase-2 (COX-2), scavenger receptor (SR)-A, and SR-B1 were purchased from Santa Cruz Biotechnology (Santa Cruz, CA, USA). CD163 antibody was supplied by Aviva system (San Diego, CA, USA). Antibodies of arginas-1, PPARγ, phospho-tyrosine kinase 2 (Tyk2), TGF-β, glucose transporter 1 (GLUT1), Akt, phospho-Akt, mammalian target of rapamycin complex (mTOR), phospho-mTOR, 5’-adenosine monophosphate-activated protein kinase (AMPK), and phospho-AMPK were obtained from Cell Signaling Technology (Danvers, MA, USA). Phospho-STAT6 antibody was provided by Thermo Fisher Scientific (Waltham, MA, USA). Connective tissue growth factor (CTGF) antibody was purchased from Pepro Tech (Rocky Hill, NJ, USA). α-Smooth muscle actin (α-SMA) antibody was obtained from Abcam (Cambridge, UK). ATP-binding cassette sub-family G member 1 (ABCG1) antibody was purchased from Novus Biological (Rockville, MD, USA). β-Actin antibody was purchased from Sigma Aldrich Chemicals. Horseradish peroxidase (HRP)-conjugated goat anti-rabbit IgG, goat anti-mouse IgG, and donkey anti-goat IgG were supplied by Jackson Immuno-Research Laboratories (West Grove, PA, USA).

### 2.2. Cell Culture

Mouse macrophages-like cell line J774A.1 (American Type Culture Collection, Manassas, VA, USA) were grown in DMEM supplemented with 10% FBS at 37 °C in a humidified atmosphere of 5% CO_2_ in air. However, in culture experiments J774A.1 macrophages were incubated in DMEM supplemented with 0.4% BSA. The macrophages were pre-treated with 1–20 μM asaronic acid and exposed to 10–50 ng/mL IL-4 for up to 72 h. In another set of experiments, J774A.1 macrophages were incubated in media containing 33 mM glucose for up to 72 h in the absence and presence of 1–20 μM asaronic acid. Previous other studies have used glucose concentration ranging from 25 to 40 mM to mimic diabetic condition in cell cultures [27,28].

Human monocytic leukemic cell line THP-1(American Type Culture Collection) was grown in HEPES-buffered RPMI 1640 containing 10% FBS, 2 mM glutamine, 100 U/mL penicillin and 0.1 mg/mL streptomycin at 37 °C in a humidified atmosphere of 5% CO_2_ in air. For the THP-1 cell differentiation, the cells were cultured in 50 ng/mL PMA-containing RPMI 1640 media. After differentiation, THP-1-derived macrophages were incubated in 0.4% BSA-added DMEM containing 33 mM glucose for 48 h in the presence of 1–20 μM asaronic acid.

The cytotoxicity of asaronic acid was determined by using MTT assay. Cells were treated with asaronic acid for 24 h in the absence and presence of IL-4 and incubated with 1 mg/mL MTT solution at 37 °C for 3 h, resulting in the formation of insoluble purple formazan product that was dissolved in 250 μL isopropanol. Optical density was measured using a microplate reader (Bio-Rad Model 550, Hercules, CA, USA) at the wavelength of 570 nm. This study found that asaronic acid and IL-4 had no cytotoxicity within the doses of 1–20 μM and 10–50 ng/mL IL-4, respectively (Figure 1B,C). Thus, the current experiments employed 1–20 μM asaronic acid and 40 ng/mL IL-4.

### 2.3. Western Blot Analysis

Following the culture protocols, the cells were lysed in a lysis buffer. Equal protein amounts of cell lysates were electrophoresed on an 8–12% sodium dodecyl sulfated-polyacrylamide gel and transferred onto a nitrocellulose membrane. After blocking with 5% skim milk or 3% BSA for 3 h at room temperature, the membranes were incubated with polyclonal or monoclonal antibodies of IL-4Rα, arginase-1, CD163, PPARγ, VEGF, PDGF, phospho-Tyk2, phospho-STAT6, GLUT1, Akt, phospho-Akt, mTOR, phospho-mTOR, AMPKα, phospho-AMPK, COX-2, CTGF, α-SMA, SR-A, SR-B1, and ABCG1 overnight at 4 °C. After three times of washing with Tris-buffered saline-tween 20, the membranes were incubated with anti-rabbit or anti-mouse IgG conjugated to HRP for 1 h. The individual protein level was detected by Immobilon Western Chemiluminescent HRP substrate (Millipore, Billerica, MA, USA). For the internal control, the membranes were incubated with β-actin antibody. After the performing immunoblot analyses, the blot bands were visualized on Agfa X-ray film (Agfa HealthCare NV, Mortsel, Belguim), developing signals with X-ray developer and fixer (Duksan, Seoul, Korea).

### 2.4. Enzyme-Linked Immunosorbent Assay (ELISA)

Cell culture media were collected from J774A.1 macrophages and stored at −20 °C. The secretion of IL-10 and IL-4 was examined in culture media by using ELISA kits (R&D System, Minneapolis, MN, USA), according to a manufacturer’s instruction.

### 2.5. Immunocytochemical Analysis

After J774A.1 macrophages were exposed to 40 ng/mL IL-4 or 33 mM glucose in the absence and presence of 1–20 μM asaronic acid for 48 h, cells were fixed with 4% formaldehyde for 15 min permeated with 0.1% Triton X-100 and 0.1% sodium citrate for 1 min on the ice. Cells were treated with 5% BSA for 1 h. For the Immunofluorescent cytochemical staining, cells were incubated with a specific primary antibody against TGF-β or phospho-STAT6 overnight and further with Cy3-conjugated or FITC-conjugated IgG for 1 h (Rockland, Pottstown, PA, USA) and washed with phosphate-buffered saline-tween 20. For the nuclear staining, cells were incubated with 4′,6-diamidino-2-phenylindole (DAPI) for 10 min. Each slide was mounted in VectaMount mounting medium (Vector Laboratories, Burlingame, CA, USA). Images were taken using an optical Axiomager microscope system (Zeiss, Oberkochen, Germany).

### 2.6. Dihydroethidium (DHE) Staining for ROS Production

The J774A.1 macrophages were plated on a 24-well glass slide and incubated with 33 mM glucose in the absence and presence of 20 μM asaronic acid for 48 h. Cells were treated with 300 μL DHE, incubated for 30 min at 37 °C, and fixed with 4% formaldehyde for 30 min at room temperature. After the fixation, the cells were treated with 0.1% Triton X-100 and 0.1% sodium citrate for 1 min on the ice. Nuclear staining was done with DAPI for 10 min. The slides were mounted in VectaMount mounting medium. Images were taken using an optical Axiomager microscope system.

### 2.7. Data Analysis

The results were expressed as mean ± SEM for each treatment group in each experiment. Statistical analyses were performed using Statistical Analysis Systems statistical software package (SAS Institute, Cary, NC, USA). Significance was determined by one-way analysis of variance, followed by Duncan range test for multiple comparisons. Differences were considered significant at *p* < 0.05.

## 3. Results

### 3.1. IL-4-Mediated IL-4Rα Induction by Asaronic Acid

The presence of IL-4 at doses of ≤50 ng/mL did not influence the macrophage viability (Figure 1C). The cell viability was not changed despite the 48 h-incubation of macrophages with 40 ng/mL IL-4 (Figure 1D). When macrophages were exposed to 40 ng/mL IL-4 for up to 72 h, the IL-4Rα expression was promptly enhanced up to 24 h and subsequently was restored to that of IL-4-untreated cells (Figure 1E). The IL-4Rα induction was further enhanced in 10 μM asaronic acid-treated macrophages (Figure 1F).

### 3.2. IL-4-Mediated M2 Macrophage Activation by Asaronic Acid

**Figure 2:**
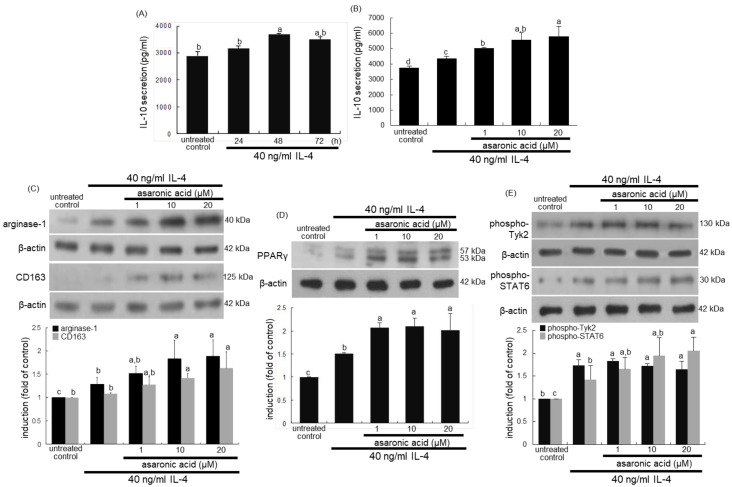
ELISA data showing temporal response and upregulation of IL-10 secretion (**A**,**B**) and Western blot data showing upregulation of expression of arginase-1, CD163, PPARγ, phospho-Tyk2, and phospho-STAT6 by asaronic acid (**C**–**E**). J774A.1 macrophages were exposed to 40 ng/mL IL-4 in the absence and presence of 1–20 μM asaronic acid for up to 48 h. For the measurements of IL-10 secretion, IL-10 in cell culture media was detected by using an ELISA kit (**A**,**B**). Cell lysates were subject to 8–12% SDS-PAGE and Western blot analysis with a primary antibody against arginase-1, CD163, PPARγ, phospho-Tyk2, and phospho-STAT6. β-Actin antibody was used as an internal control. The bar graphs (mean ± SEM, *n* = 3) in the bottom panels represent quantitative results of the upper blot bands obtained from a densitometer. Mean values in respective bar graphs not sharing a same lower-case alphabet letter are significantly different at *p* < 0.05.

This study examined whether asaronic acid elevated IL-4-mediated macrophage polarization. Th1 cytokines promote monocyte differentiation into M1 macrophages, while Th2 cytokines lead to an alternative anti-inflammatory M2 macrophage phenotype [2]. The production of the Th2 cytokine IL-10 was elevated by exposure of macrophages to IL-4 for ≥48 h, as evidenced by ELISA (Figure 2A). When macrophages were exposed to 40 ng/mL IL-4 for 48 h, 1–20 μM asaronic acid further increased the secretion of IL-10, a M2 marker (Figure 2B). Western blot data showed that 40 ng/mL IL-4 highly increased the induction of the M2 markers of arginase-1 and CD163, which was further augmented by ≥10 μM asaronic acid (Figure 2C). Accordingly, asaronic acid promoted macrophage polarization toward the M2 phenotype possibly via the IL-4-IL-4 Rα interaction.

PPARγ is a ligand-activated nuclear receptor with potent anti-inflammatory properties that modulates the immune inflammatory response, and its activation primes human monocytes into alternative M2 macrophages [29]. This study attempted to determine whether asaronic acid stimulated the PPAR signaling for the M2 macrophage polarization mediated by IL-4. As expected, the PPARγ induction was highly boosted in macrophages exposed to IL-4 (Figure 2D). Such induction was further elevated in ≥1 μM asaronic acid-administrated macrophages. In addition, the IL-4 presence prompted the tyrosine phosphorylation and STAT6 activation in macrophages (Figure 2E). STAT6, known to drive macrophage M2 polarization, is a facilitator of the nuclear receptor PPARγ-regulated gene expression in macrophages and dendritic cells [30]. Asaronic acid further accelerated the activation of Tyk2 and STAT6 in IL-4-exposed macrophages (Figure 2E). These results indicate that asaronic acid may lead to the induction of anti-inflammatory M2 genes targeted by PPARγ.

### 3.3. Angiogenic and Proliferative Capacity of Asaronic Acid in M2-Polarized Macrophages

**Figure 3:**
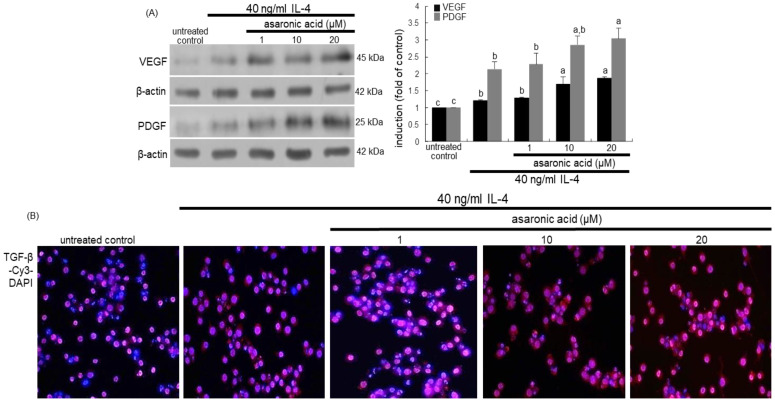
Elevation of induction of PDGF, VEGF, and TGF-β by asaronic acid. J774A.1 macrophages were exposed to 40 ng/mL IL-4 in the absence and presence of 1–20 μM asaronic acid for 48 h. Cell lysates were subject to 8–12% SDS-PAGE and Western blot analysis with a primary antibody against PDGF and VEGF (**A**). β-Actin antibody was used as an internal control. The bar graphs (mean ± SEM, *n* = 3) in the panels represent quantitative results of the left blot bands obtained from a densitometer. Mean values in respective bar graphs not sharing a same lower-case alphabet letter are significantly different at *p* < 0.05. Immunocytochemical analysis showing TGF-β induction of IL-4-treated macrophages (**B**). The TGF-β localization was confirmed by Cy3-red staining in macrophages exposed to IL-4 (n=3). Nuclear staining was done with DAPI (blue). Magnification: 200-fold.

Macrophages involved in pro-wound healing process produce elevated levels of growth factors such as PDGF, insulin-like growth factor 1, VEGF, and TGF-β1, which facilitate cellular proliferation, tissue formation, and angiogenesis [9,10]. The current study attempted to examine whether asaronic acid elevated angiogenic and proliferative capacity of M2-polarized macrophages. When IL-4 was added to macrophages for 48 h, the induction of VEGF and PDGF was markedly enhanced (Figure 3A). Moreover, the co-treatment of ≥1 μM asaronic acid with IL-4 promoted the induction of these growth factors in a dose-dependent manner. Additionally, there was a strong fluorescent staining of Cy3-TGF-β in asaronic acid-bearing macrophages, indicating marked induction of TGF-β by asaronic acid (Figure 3B).

### 3.4. Effects of Asaronic Acid on IL-4-Mediated M2 Macrophage Metabolism

**Figure 4:**
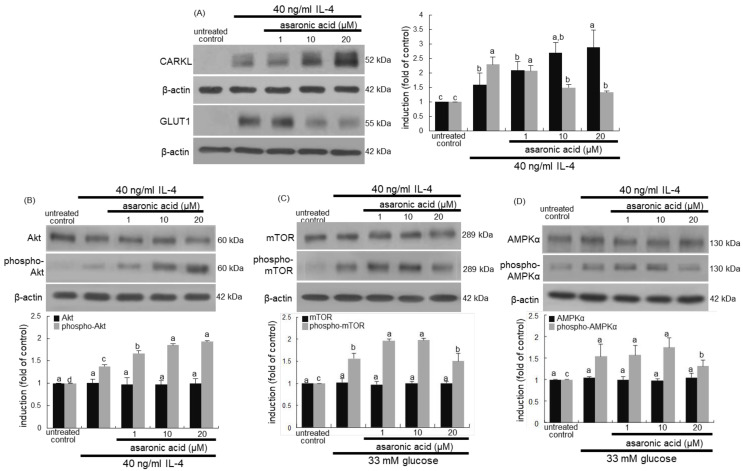
Western blot analysis showing effects of asaronic acid on induction of CARKL, GLUT1, phospho-Akt, phospho-mTOR, and phospho-AMPKα (**A**–**D**). J774A.1 macrophages were exposed to 40 ng/mL IL-4 in the absence and presence of 1-20 μM asaronic acid for 48 h. Cell lysates were subject to 8–12% SDS-PAGE and Western blot analysis with a primary antibody against CARKL, GLUT1, Akt, phospho-Akt, mTOR, phospho-mTOR, AMPKα, and phospho-AMPKα. β-Actin antibody was used as an internal control. The bar graphs (mean ± SEM, *n* = 3) in the panels represent quantitative results of the blot bands obtained from a densitometer. Mean values in respective bar graphs not sharing a same lower-case alphabet letter are significantly different at *p* < 0.05.

Metabolic reprogramming of activated macrophages is required for proper polarization and function [31]. This study examined whether asaronic acid favorably manipulated metabolic reprogramming in IL-4-activated M2 macrophages. The IL-4 treated to macrophages enhanced the induction of CARKL involved in the metabolic process for macrophage polarization, in which ≥10 μM asaronic acid further augmented the CARKL induction (Figure 4A). On the other hand, the glucose transporter GLUT1induction was highly enhanced in response to IL-4, but treating ≥10 μM asaronic acid attenuated such induction (Figure 4A). It was assumed that asaronic acid diminished cellular glucose availability and glycolysis.

Metabolic signaling pathways of macrophages activated by polarizing signals include Akt, mTOR complex, and AMPK [32]. This study investigated that asaronic acid affected metabolic signaling pathways in IL-4-polarized M2 macrophages. IL-4 augmented Akt activation during M2 macrophage activation, which was further prompted by asaronic acid (Figure 4B). In addition, the phosphorylation of mTOR and AMPKα was promoted in IL-4-exposed macrophages during M2 activation (Figure 4C,D). Such IL-4-induced phosphorylation was further enhanced in asaronic acid-added macrophages. Accordingly, asaronic acid may be a contributing upstream activator of Akt, mTOR, and AMPK in parallel with the IL-4Rα-Tyk2-STAT6 pathway through reducing glucose uptake.

### 3.5. Inhibition of Glucose-Triggered M2 Macrophage Activation by Asaronic Acid

**Figure 5:**
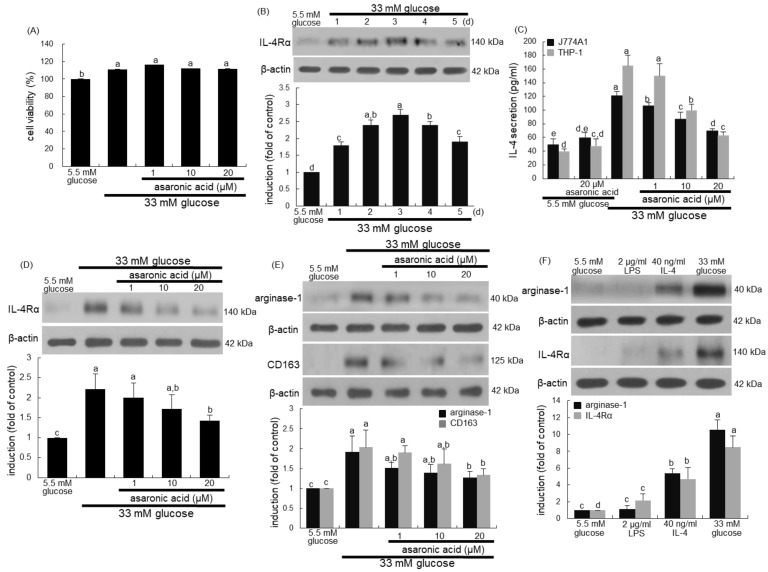
Cell viability of asaronic acid (**A**), temporal response of IL-4Rα induction to glucose (**B**), inhibition of IL-4 secretion and induction of IL-4Rα, arginase-1, and CD163 by asaronic acid (**C**–**E**) and induction of arginase-1 and IL-4Rα by 5.5 mM glucose, 2 μg/mL LPS, 40 ng/mL IL-4, and 33 mM glucose (**F**). J774A.1 macrophages were exposed to 33 mM glucose in the absence and presence of 1–20 μM asaronic acid for up to 5 days. THP-1 monocytes were differentiated to macrophages with PMA for 5 days and then exposed to 5.5 mM or 33 mM glucose in the absence and presence of 1-20 μM asaronic acid for 48 h. Macrophage viability (mean ± SEM, *n* = 5) was measured by using MTT assay and expressed as percent cell survival relative to untreated control (**A**). For the measurements of IL-4 secretion, IL-4 in cell culture media was detected by using an ELISA kit (**C**). J774A.1 macrophages were incubated in a media containing 5.5 mM glucose, 2 μg/mL LPS, 40 ng/mL IL-4, and 33 mM glucose for 48 h (**F**). Cell lysates were subject to 8–12% SDS-PAGE and Western blot analysis with a primary antibody against IL-4Rα, arginase-1, and CD163 (**B**,**D**–**F**). β-Actin antibody was used as an internal control. The bar graphs (mean ± SEM, *n* = 3) in the panels represent quantitative results of the upper blot bands obtained from a densitometer. Mean values in respective bar graphs not sharing a same lower-case alphabet letter are significantly different at *p* < 0.05.

The present study investigated that J774A.1 macrophages were polarized toward M2 phenotype due to proliferative glucose (Figure 5A). Glucose enhanced the IL-4Rα expression in a temporal fashion with a high induction at the day 3 (Figure 5B). The IL-4 secretion was apparent in glucose-loaded J774A.1 murine macrophages and THP-1-derived human macrophages, but its secretion was inhibited by treating asaronic acid to these cells (Figure 5C). The IL-4 secretion was also enhanced in 25 mM glucose-loaded J774A.1 murine macrophages, and its secretion was much higher in 33 mM glucose-exposed macrophages (unpublished data). Accordingly, the glucose concentration ranging from 25 to 33 mM can be used in order to mimic diabetic condition in cell cultures. Asaronic acid per se did not influence the IL-4 secretion of both macrophages significantly (Figure 5C). Consistently, 1–20 μM asaronic acid dose-dependently curtailed the concurrent increase in the IL-4Rα induction by glucose (Figure 5D). These results showed that asaronic acid deterred glucose-stimulated induction of IL-4-producing M2 macrophages.

M2 macrophages polarized by glucose simultaneously increased the expression of the M2 phenotypic markers of arginase-1 and CD163 (Figure 5E). When 1–20 μM asaronic acid was administrated to glucose-loaded macrophages, expression of these proteins was highly abrogated. In another set of experiments, the relative induction levels of arginase-1 and IL-4Rα were examined in lipopolysaccharide (LPS)-, IL-4- or glucose-exposed macrophages (Figure 5F). LPS did not induce the M2 marker of arginase-1 in macrophages despite a minimal induction of IL-4Rα, compared to that of low glucose (5.5 mM)-treated macrophages. However, the induction of arginase-1 and IL-4Rα in high glucose (33 mM)-loaded macrophages was almost twice higher than that of IL-4-exposed macrophages (Figure 5F). Accordingly, glucose was a powerful inducer skewing naïve macrophages toward M2 phenotypes.

**Figure 6:**
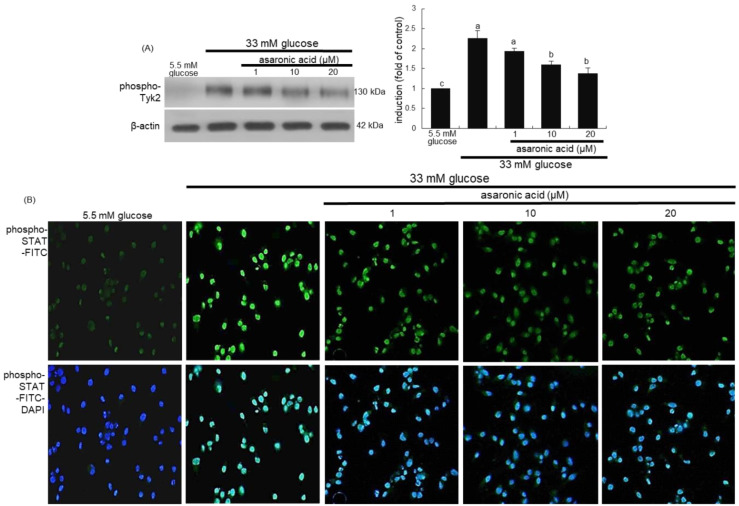
Blockade of induction of phospho-Tyk2 and phospho-STAT6 by asaronic acid. J774A.1 macrophages were exposed to 33 mM glucose in the absence and presence of 1–20 μM asaronic acid for 48 h. Cell lysates were subject to 8–12% SDS-PAGE and Western blot analysis with a primary antibody against phospho-Tyk2 (**A**). β-Actin antibody was used as an internal control. The bar graphs (mean ± SEM, *n* = 3) in the panels represent quantitative results of the left blot bands obtained from a densitometer. Mean values in respective bar graphs not sharing a same lower-case alphabet letter are significantly different at *p* < 0.05. Immunocytochemical analysis showing phospho-STAT6 induction in glucose-loaded macrophages (**B**). The phospho-STAT6 localization was confirmed by FITC-green staining in macrophages exposed to 33 mM glucose (*n* = 3). Nuclear staining was done with DAPI (blue). Magnification: 200-fold.

The glucose load stimulated the Tyk2 phosphorylation in M2 macrophages expressing IL-4Rα and arginase-1 (Figure 6A). In addition, immunocytochemical data showed a strong FITC-green phospho-STAT6 staining in glucose alone-treated macrophages, indicating that glucose evoked the STAT6 activation (Figure 6B). When asaronic acid was treated to glucose-loaded macrophages, the activation of Tyk2 and STAT6 was dose-dependently attenuated (Figure 6A,B). These results indicated that asaronic acid may hamper glucose-triggered macrophage M2 polarization through disturbing Tyk2-STAT6 signaling.

### 3.6. Glucose-Triggered Metabolic Alterations in M2 Macrophages

**Figure 7:**
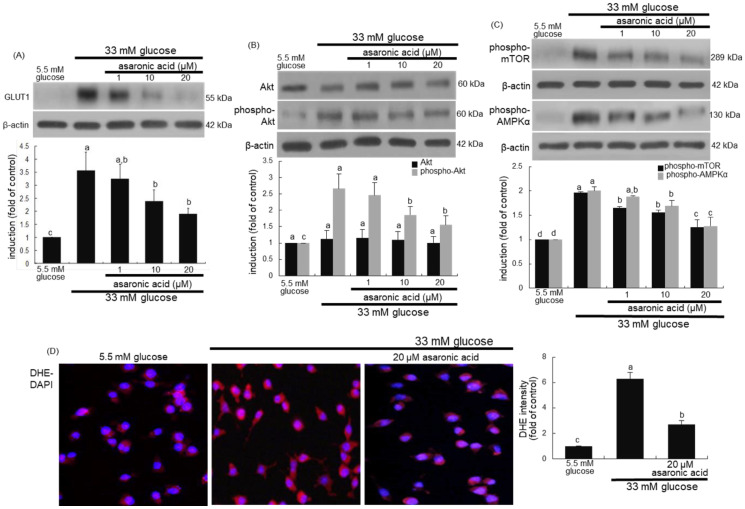
Inhibition of induction of GLUT1, phospho-Akt, phospho-mTOR, and phospho-AMPKα by asaronic acid (**A**–**C**), and blockade of ROS production by 20 μM asaronic acid (**D**). J774A.1 macrophages were exposed to 33 mM glucose in the absence and presence of 1-20 μM asaronic acid for 48 h. Cell lysates were subject to 8–12% SDS-PAGE and Western blot analysis with a primary antibody against GLUT1, Akt, phospho-Akt, phospho-mTOR, and phospho-AMPKα. β-Actin antibody was used as an internal control. The bar graphs (mean ± SEM, *n* = 3) in the panels represent quantitative results of the upper blot bands obtained from a densitometer. Mean values in respective bar graphs not sharing a same lower-case alphabet letter are significantly different at *p* < 0.05. For the measurement of ROS production (**D**), reddish DHE staining was conducted in J774A.1 macrophages exposed to 33 mM glucose in the presence of 20 μM asaronic acid. Nuclear staining was done with DAPI (blue). Magnification: 200-fold.

This study investigated that glucose stimulated GLUT1, leading to glucose uptake and sustained M2 activation of macrophages. As expected, the induction of GLUT1 was highly enhanced in glucose-exposed macrophages for M2 macrophage polarization (Figure 7A). When asaronic acid was supplied to glucose-loaded macrophages, such induction was dose-dependently reduced. Accordingly, asaronic acid may retard metabolic burden of glucose to weaken glucose-evoked M2 activation.

This study further investigated that asaronic acid attenuated glucose-triggered metabolic signaling pathways in macrophages. Glucose markedly activated Akt, mTOR, and AMPKα during M2 activation of macrophages (Figure 7B,C). In contrast, asaronic acid encumbered the activation of these metabolic targets in glucose-exposed macrophages. Thus, asaronic acid may disrupt the formation of a coordinated signaling module that was activated by glucose uptake.

Several reports have shown that ROS is involved in activation of AMPK-mTOR pathway [33,34]. This study examined whether high glucose enhanced ROS production in macrophages, which was attenuated by asaronic acid. A strong cytosolic staining of DHE was observed in glucose-loaded macrophages (Figure 7D). However, the intensity of red-DHE staining was diminished in 20 μM asaronic acid-treated diabetic macrophages. Accordingly, diabetes-associated oxidative stress may activate the Akt-mTOR-AMPK signaling pathway in macrophages, which can be disturbed by the antioxidant asaronic acid.

### 3.7. Blockade of Diabetic M2 Macrophage Dysfunction by Asaronic Acid

**Figure 8:**
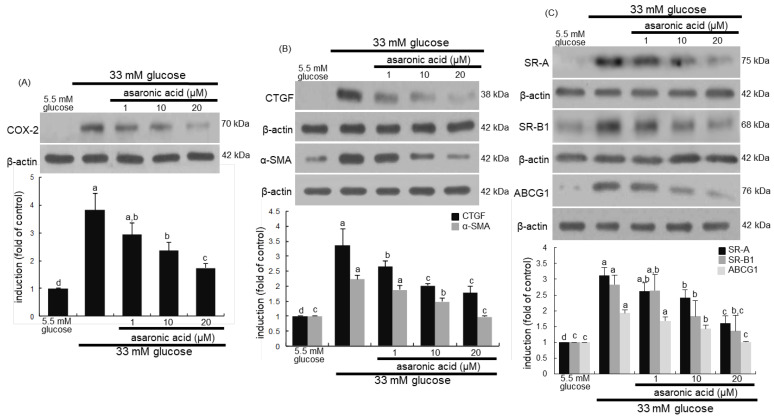
Western blot analysis showing inhibition of induction of COX-2, CTGF, α-SMA, SR-A SR-B1, and ABCG1 by asaronic acid (**A**–**C**). J774A.1 macrophages were exposed to 33 mM glucose in the absence and presence of 1–20 μM asaronic acid for 48 h. Cell lysates were subject to 8–12% SDS-PAGE and Western blot analysis with a primary antibody against COX-2, CTGF, α-SMA, SR-A, SR-B1, and ABCG1. β-Actin antibody was used as an internal control. The bar graphs (mean ± SEM, *n* = 3) in the panels represent quantitative results of the upper blot bands obtained from a densitometer. Mean values in respective bar graphs not sharing a same lower-case alphabet letter are significantly different at *p* < 0.05.

This study examined whether asaronic acid curtailed the COX-2 induction in diabetic macrophages with M2 phenotype polarity. One study shows that COX-2 in tumor-associated macrophages enhances cancer cell survival by eliciting a positive-feedback loop between macrophages and cancer cells [35]. Glucose-polarized M2 macrophages simultaneously induced COX-2 (Figure 8A), which in turn promoted macrophage proliferation (Figure 5A). In contrast, asaronic acid diminished the COX-2 induction in diabetic macrophages. These results indicate that inhibiting Akt signaling in diabetic macrophages abrogated COX-2 expression and macrophage proliferation (Figure 7B).

M2 macrophages enhance myofibroblast differentiation of lung resident mesenchymal stem cells, and they are associated with pulmonary fibrogenesis [36]. The exacerbation of fibrosis depends on macrophage polarization and persistence of adverse insults [37]. This study investigated that asaronic acid dampened the induction of tissue fibrotic factors elevated by glucose insult. The induction of the fibrotic CTGF and α-SMA was promoted in M2-polarizing diabetic macrophages (Figure 8B). High glucose may play an important role in developing fibrosis through up-regulation of expression of TGF-β and CTGF [37]. However, asaronic acid dose-dependently reduced such induction in diabetic M2 macrophages.

Expression of SR-A is enhanced by high glucose and under diabetic conditions, which is one mechanism for an increased rate of atherosclerosis in diabetes [38]. In addition to the scavenger receptor CD163, the induction of SR- A and SR-B1 was evaluated in macrophages treated with glucose in the presence of asaronic acid. Interestingly, the polarity and phenotypic transformation of macrophages affects cholesterol homeostasis such as cholesterol uptake, efflux and storage [39]. M2-polarizing diabetic macrophages markedly induced SR-A, SR-B1, and ABCG1 (Figure 8C), which could be responsible for diabetes-associated pathogenesis of atherosclerosis and inflammation. On the other hand, 1–20 μM asaronic acid blocked the induction of both SR protein and ABCG1 in glucose-loaded macrophages in a dose dependent manner.

## 4. Discussion

Eight major findings were observed from this study. (1) The IL-4Rα induction and IL-10 secretion by IL-4 in macrophages were further promoted by the presence of asaronic acid. (2) Submicromolar asaronic acid augmented cellular induction of arginase-1, CD163, PPARγ, VEGF, PDGF, and TGF-β of macrophages elevated by IL-4. (3) IL-4 enhanced the induction of CARKL and GLUT1 in macrophages, which was reciprocally manipulated by asaronic acid. (4) The activation of Tyk2, STAT6, Akt, mTOR, and AMPKα was highly boosted in M2-polarizing macrophages by co-treatment of IL-4 and asaronic acid. (5) Asaronic acid curtailed the IL-4 secretion and IL-4Rα induction in glucose-loaded macrophages. (6) Induction of arginase-1 and CD163 by glucose was highly abrogated in asaronic acid-treated macrophages. (7) Asaronic acid encumbered the activation of Tyk2, STAT6, Akt, mTOR, and AMPKα as well as GLUT1 induction in glucose-loaded macrophages. (8) Asaronic acid abrogated the induction of the inflammatory COX-2, the fibrotic CTGF and α-SMA, and the scavenging SR-A and SR-B1in diabetic macrophages with M2 phenotype polarity. Together, asaronic acid promoted macrophage polarization toward the M2 phenotype by IL-4-IL-4Rα interaction, leading to elevation of angiogenic and proliferative capacity of macrophages. On the contrary, asaronic acid deterred macrophage polarization toward the M2 phenotype in diabetic macrophages through disrupting the formation of a coordinated signaling of GLUT1-Akt-mTOR-AMPKα activated by glucose uptake.

Macrophage polarization is closely regulated via activation of diverse signaling pathways under physiologic and pathologic conditions [1,2]. A critical role in plasticity and heterogeneity of macrophages depends on activation of STAT downstream signaling [1,3]. In contrast to STAT1 activation of M1 macrophage polarization by LPS and interferon (IFN)-γ, the STAT3/ STAT6 activation is involved in induction of M2 macrophage polarization by IL-4, IL-13, IL-10, and PPARγ [4,6,7]. Another report shows that shifting into immunosuppressive mesenchymal stem cell 2-phenotype entails IFN-γ-mediated interplay of STAT1 and phosphoinositide 3-kinases for induction of immunoregulatory indoleamine 2,3-dioxygenase [40]. Macrophage polarization toward a M2 phenotype leads to anti-inflammation, tissue remodeling, vasculogenesis, and injury repair in a damaged organ [4,6,7]. Anti-inflammatory properties of M2 macrophages are important in resolving the inflammation through producing the immunosuppressive cytokine IL-10 [8,41]. Wound healing of macrophages upregulates IL-4 production and arginase activity, thus regenerating the damaged tissues [4]. Several studies have shown therapeutic potentials of pharmacological agents for the treatment of tissue injury via targeting macrophage polarization [18,20]. Use of naturally occurring compounds have been proposed as a therapeutic strategy targeting macrophage polarization [21,22]. This study examined whether submicromolar asaronic acid manipulated macrophage polarization in a positive way by treating IL-4 to macrophages. Asaronic acid further accelerated the macrophage induction of IL-4Rα, arginase-1, CD163, and PPARγ as well as the IL-10 production enhanced by IL-4, indicating that this compound activated macrophages toward an induction of M2 phenotype. In addition, asaronic acid further enhanced the induction of growth factors of VEGF, PDGF, and TGF-β in IL-4-treated macrophages. These findings indicate that asaronic acid aided in cellular proliferation, tissue formation, and angiogenesis of M2-polarizing macrophages.

An imperative role of macrophage polarization has become increasingly implicated in the development of metabolic diseases including obesity and type 2 diabetes [11,12]. In general, there is an imbalance of macrophage polarization toward increase in M1 macrophages, leading to chronic inflammation and the propagation of metabolic dysfunction [11]. However, macrophage polarization is much complicated in obesity and type 2 diabetes owing to different macrophage profiles beyond the M1/M2 binary classification. The current study examined whether high glucose evoked macrophage polarization toward a M2 phenotype. Similar to IL-4 and IL-13, glucose induced IL-4-producting M2 macrophages with a marked expression of IL-4Rα and arginase-1. In fact, the induction of IL-4Rα and arginase-1 was much higher in glucose-inflamed M2 macrophages than in IL-4-alone-triggered M2 macrophages. Thus, it can be assumed that glucose-induced macrophages responded to the IL-4 in an autocrine fashion. In our previous study, high glucose induced macrophage activation toward the M1 phenotype, which led to diabetic inflammation through triggering toll-like receptor 4-mediated NF-κB/JAK2-STAT signaling [26]. Together, macrophages exposed to glucose showed both M1 and M2 phenotypes, indicating that both M1 and M2 macrophages may coexist, possibly resulting in persistent inflammation, fibrosis, and atherogenesis that are common features in diabetes [42]. Metabolic aberration to high glucose may instruct activation of macrophages with functionally distinct M2 phenotypes, which in turn probably leads to the pathogenesis of diabetes-associated diseases.

Several studies have shown that natural compounds especially prompt macrophage polarization toward induction of M2 phenotype via diverse molecular mechanisms [23,24,43]. To alleviate dextran sodium sulphate-induced experimental colitis, the soya genistein induces macrophage polarization of M1 towards M2 macrophages and reduces systemic cytokines [24]. In addition, quercetin modulates synovial macrophages polarization to M2 macrophages, hence creating a pro-chondrogenic microenvironment for chondrocytes to enhance cartilage repair under osteoarthritis condition [44]. Accordingly, these natural compounds may boost the induction of macrophage polarization with M2 phenotype in the pathogenesis of infectious and autoimmune diseases. On the other hand, corosolic acid inhibits the M2 polarization of macrophages and tumor cell proliferation by inhibiting both STAT3 and NF-κB activation [43]. Furthermore, dietary flavonoid isoliquiritigenin inhibits colitis-triggered tumorigenesis through blocking M2 macrophage polarization mediated by the interplay of prostaglandin E2 and IL-6 [45]. Similarly, asaronic acid led to inhibition of glucose-induced M2 macrophage phenotype in parallel with reducing expression of arginase-1 and CD163. This study attempted to reveal that asaronic acid manipulated macrophage plasticity in a proper and precise way in the diabetic pathogenesis. These findings indicate that by glucose-primed macrophage polarization toward M2 phenotypes may be detrimental in diabetes and cancer.

Since there is not a rigid barrier between macrophage phenotypes [8], excessive function of M2 macrophages by glucose would be unfavorable. In general, M2 macrophages contribute to function in constructive processes such as tissue repair, remodeling, and vasculogenesis; deactivate damaging immune activation; and retain homeostasis [1,11]. Quercetin accelerates diabetic wound healing via switching macrophages from M1 to M2 polarization [46]. Although M2 macrophages play crucial roles in determining short-term recovery, they could be a main culprit in the development of fibrotic diseases. This study found that asaronic acid suppressed the glucose induction of COX-2, CTGF, and α-SMA responsible for tissue remodeling and fibrosis-tropic process. Accordingly, asaronic acid may hamper diabetes-associated fibrotic disorders apparent in organs such as kidney and liver through reducing fibrotic mediators during M2 macrophage activation. Better strategies and targets to induce reparative macrophages in vivo should guide future investigations in order to abate fibrotic diseases. On the other hand, expression of SR-A and CD36 enhanced by high glucose is known to be one mechanism for an increased rate of atherosclerosis in diabetes [38,47]. In addition, it has been reported that ABCG1 modulates M2 macrophage polarization in macrophages under pathological conditions including cancer, obesity and caloric restriction [48,49]. Asaronic acid inhibited the enhanced induction of SR-A and SR-B1 during M2 macrophage activation by glucose. In contrast, ginsenoside Rg3 reduces diabetic atherosclerosis by skewing macrophages to M2 phenotype [50]. Unfortunately, this study cannot answer the discrepancy. It is important to note that M1 and M2 macrophages may be simultaneously present at the same location, and that the dominance of M1 or M2 may be the key factor influencing disease progression [51]. However, the diversity of macrophage phenotypes in vivo remains to be fully characterized.

Recent advances in transcriptomic and metabolomic studies have highlighted the crosstalk between metabolic rewiring of macrophages and their functional plasticity [52,53]. Proper polarization and function of macrophages require metabolic reprogramming via signaling pathways including Akt, mTOR complex, and AMPK [31,32,53]. Asaronic acid had positive impacts on metabolic reprogramming creating enhanced induction and activation of CARKL, Akt, mTOR, and AMPKα in IL-4-activated M2 macrophages. In contrast, asaronic acid encumbered activation of these metabolic targets in glucose-loaded macrophages. This could be a possible mechanism for inhibition of glucose-induced M2 activation of macrophages by the antioxidant asaronic acid attenuating ROS production. Similarly, resveratrol prevents palmitic acid-induced intracellular ROS by autophagy regulation via the AMPK-mTOR pathway [54]. Furthermore, asaronic acid attenuated cellular uptake and availability of glucose through diminishing GLUT1 induction in response to glucose. Especially, asaronic acid may disrupt the formation of a coordinated metabolic signaling module evoking diabetic reprogramming in parallel with the IL-4Rα-Tyk2-STAT6 pathway. Accordingly, asaronic acid may retard metabolic burden of glucose on GLUT1-Akt-mTOR-AMPKα to diminish M2 activation.

## 5. Conclusions

The current study investigated the capability of asaronic acid in manipulating macrophage activation toward induction of M2 phenotype in IL-4-exposed or glucose-loaded macrophages. Nontoxic asaronic acid accelerated macrophage activation toward M2 phenotype through inhibition of IL-4Rα-mediated signaling involving Tyk2-STAT6 pathway. Asaronic acid aided in cellular proliferation, tissue formation, and angiogenesis of macrophages through enhancing growth factors of VEGF, PDGF, and TGF-β. On the contrary, asaronic acid attenuated glucose-triggered M2 macrophage activation with a concomitant inhibition of IL-4Rα-Tyk2-STAT6 signaling and GLUT1-Akt-mTOR-AMPKα pathway. Asaronic acid abrogated induction of COX-2, CTGF/α-SMA, and SR-A/SR-B1 by glucose during M2 macrophage activation. Accordingly, asaronic acid may inhibit diabetic macrophage dysfunction due to M2 activation through disturbing IL-4Rα-mediated M2 gene expression and GLUT1-triggered metabolic aberration. However, the current study still requires further investigation on the effect of asaronic acid on macrophage glucose uptake after stimulation with insulin and on Akt phosphorylation upon stimulation with insulin.

## Abbreviations

AMPK, 5’-adenosine monophosphate-activated protein kinase; CARKL, carbohydrate kinase-like, COX-2, cyclooxygenase-2; CTGF, connective tissue growth factor; GLUT; glucose transporter; JAK, Janus kinase; IL, interleukin; IL-4Rα, IL-4 receptor Rα; LPS, lipopolysaccharide; mTOR, mammalian target of rapamycin; NF-κB, nuclear factor-κB; PDGF, platelet-derived growth factor; PPARγ, peroxisome proliferator-activated receptor γ; α-SMA, α-smooth muscle actin; SR, scavenger receptor; STAT, signal transducer and activator of transcription; TGF-β, transforming growth factor-β; Tyk2, tyrosine kinase 2; VEGF, vascular endothelial growth factor.
